# Construction of a map-based reference genome sequence for barley, *Hordeum vulgare* L.

**DOI:** 10.1038/sdata.2017.44

**Published:** 2017-04-27

**Authors:** Sebastian Beier, Axel Himmelbach, Christian Colmsee, Xiao-Qi Zhang, Roberto A. Barrero, Qisen Zhang, Lin Li, Micha Bayer, Daniel Bolser, Stefan Taudien, Marco Groth, Marius Felder, Alex Hastie, Hana Šimková, Helena Staňková, Jan Vrána, Saki Chan, María Muñoz-Amatriaín, Rachid Ounit, Steve Wanamaker, Thomas Schmutzer, Lala Aliyeva-Schnorr, Stefano Grasso, Jaakko Tanskanen, Dharanya Sampath, Darren Heavens, Sujie Cao, Brett Chapman, Fei Dai, Yong Han, Hua Li, Xuan Li, Chongyun Lin, John K. McCooke, Cong Tan, Songbo Wang, Shuya Yin, Gaofeng Zhou, Jesse A. Poland, Matthew I. Bellgard, Andreas Houben, Jaroslav Doležel, Sarah Ayling, Stefano Lonardi, Peter Langridge, Gary J. Muehlbauer, Paul Kersey, Matthew D. Clark, Mario Caccamo, Alan H. Schulman, Matthias Platzer, Timothy J. Close, Mats Hansson, Guoping Zhang, Ilka Braumann, Chengdao Li, Robbie Waugh, Uwe Scholz, Nils Stein, Martin Mascher

**Affiliations:** 1 Leibniz Institute of Plant Genetics and Crop Plant Research (IPK) Gatersleben, 06466 Seeland, Germany; 2 School of Veterinary and Life Sciences, Murdoch University, Murdoch, Western Australia 6150, Australia; 3 Centre for Comparative Genomics, Murdoch University, Murdoch, Western Australia 6150, Australia; 4 Australian Export Grains Innovation Centre, South Perth, Western Australia 6151, Australia; 5 Department of Agronomy and Plant Genetics, University of Minnesota, St Paul, Minnesota 55108, USA; 6 The James Hutton Institute, Dundee DD2 5DA, UK; 7 European Molecular Biology Laboratory—The European Bioinformatics Institute, Hinxton CB10 1SD, UK; 8 Leibniz Institute on Aging—Fritz Lipmann Institute (FLI), 07745 Jena, Germany; 9 BioNano Genomics Inc., San Diego, California 92121, USA; 10 Institute of Experimental Botany, Centre of the Region Haná for Biotechnological and Agricultural Research, 78371 Olomouc, Czech Republic; 11 Department of Botany & Plant Sciences, University of California, Riverside, Riverside, California 92521, USA; 12 Department of Computer Science and Engineering, University of California, Riverside, Riverside, California 92521, USA; 13 Department of Agricultural and Environmental Sciences, University of Udine, 33100 Udine, Italy; 14 Green Technology, Natural Resources Institute (Luke), Viikki Plant Science Centre, and Institute of Biotechnology, University of Helsinki, 00014 Helsinki, Finland; 15 Earlham Institute, Norwich NR4 7UH, UK; 16 BGI-Shenzhen, Shenzhen 518083, China; 17 College of Agriculture and Biotechnology, Zhejiang University, Hangzhou 310058, China; 18 Kansas State University, Wheat Genetics Resource Center, Department of Plant Pathology and Department of Agronomy, Manhattan, Kansas 66506, USA; 19 School of Agriculture, University of Adelaide, Urrbrae, South Australia 5064, Australia; 20 Department of Plant and Microbial Biology, University of Minnesota, St Paul, Minnesota 55108, USA; 21 School of Environmental Sciences, University of East Anglia, Norwich NR4 7UH, UK; 22 National Institute of Agricultural Botany, Cambridge CB3 0LE, UK; 23 Department of Biology, Lund University, 22362 Lund, Sweden; 24 Carlsberg Research Laboratory, 1799 Copenhagen, Denmark; 25 Department of Agriculture and Food, Government of Western Australia, South Perth, Western Australia 6150, Australia; 26 Hubei Collaborative Innovation Centre for Grain Industry, Yangtze University, Jingzhou, Hubei 434025, China; 27 School of Life Sciences, University of Dundee, Dundee DD2 5DA, UK; 28 School of Plant Biology, University of Western Australia, Crawley 6009, Australia; 29 German Centre for Integrative Biodiversity Research (iDiv) Halle-Jena-Leipzig, 04103 Leipzig, Germany

**Keywords:** DNA sequencing, Plant genetics, Genome assembly algorithms

## Abstract

Barley (*Hordeum vulgare* L.) is a cereal grass mainly used as animal fodder and raw material for the malting industry. The map-based reference genome sequence of barley cv. ‘Morex’ was constructed by the International Barley Genome Sequencing Consortium (IBSC) using hierarchical shotgun sequencing. Here, we report the experimental and computational procedures to (i) sequence and assemble more than 80,000 bacterial artificial chromosome (BAC) clones along the minimum tiling path of a genome-wide physical map, (ii) find and validate overlaps between adjacent BACs, (iii) construct 4,265 non-redundant sequence scaffolds representing clusters of overlapping BACs, and (iv) order and orient these BAC clusters along the seven barley chromosomes using positional information provided by dense genetic maps, an optical map and chromosome conformation capture sequencing (Hi-C). Integrative access to these sequence and mapping resources is provided by the barley genome explorer (BARLEX).

## Background & Summary

Barley (*Hordeum vulgare* L.) is a cereal grass of great agronomical importance. The goal of the International Barley Genome Sequencing Consortium (IBSC) is the construction of a map-based reference sequence assembly of barley cultivar ‘Morex’ by means of hierarchical shotgun sequencing^1^. Towards this aim, the barley genomics community has developed an array of genome-wide physical and genetic mapping resources. These include libraries of bacterial artificial chromosomes (BACs)^2^, a genome-wide physical map^3^, a draft whole genome shotgun (WGS) assembly^4^ and an ultra-dense genetic map^5^. The last stage on the road towards the reference genome is the shotgun sequencing of BAC clones along a minimum tiling path of the genome defined by the physical map. The advances in high-throughput sequencing technology enabled this task to be completed in a much shorter timeframe than was required for the completion of, for instance, the human^6^ and maize^7^ genomes. In addition to the generation of BAC raw sequence data, we constructed (i) physical genome maps by single-molecule optical mapping in nanochannels^8^ and by chromosome conformation capture sequencing (Hi-C)^9,10^, and (ii) a high-resolution genetic map of a large bi-parental mapping population through genotyping-by-sequencing^11^. We undertook the sequence assembly of individual BACs, the construction of larger sequence scaffolds by merging sequences from adjacent clones and the integration of these super-scaffolds with the various genome-wide mapping resources constructed in the present effort as well as those published previously^3,5^. The final outcome of this approach was the construction of ‘pseudomolecules’, i.e., contiguous sequence scaffolds representing the seven chromosomes of barley.

We have submitted the relevant raw data to public sequence data archives, made analysis results available under permanent digital object identifiers (DOIs) and entered the positional information used for pseudomolecule construction into a bespoke information management system, the BARLEX genome explorer^12^. Here, we give (i) a comprehensive overview of datasets used for assembling the barley genome and methods employed in their generation, (ii) a detailed description of wet-lab procedures for BAC sequencing and the bioinformatics workflow of the sequence assembly and data integration procedures together with an outline of (iii) their browsable presentation in an online database. These resources document the construction of the map-based reference sequence of the barley genome and will enable researchers to inspect the evidence used to assemble, order and orient sequence scaffolds and may guide the further improvement of the genome sequence with complementary data sets.

## Methods

**Figure 1:**
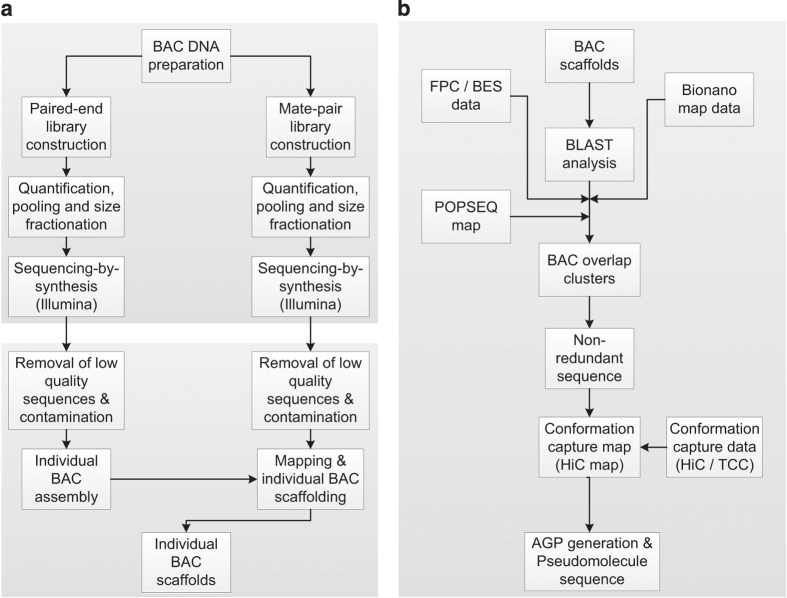
Assembly workflow. **(a)** Assembly of individual BAC clones from paired-end and mate-pair read data. **(b)** Data integration procedures for pseudomolecule construction.

The main steps for the construction of the map-based reference sequence of the barley genome were (i) shotgun and mate-pair sequencing of BAC clones, (ii) sequence assembly of individual BAC clones and (iii) the construction of a pseudomolecule sequences by merging the sequences of adjacent BACs into super-scaffolds and ordering these using various sources of positional information such as physical maps, optical map and chromosome conformation capture. A schematic overview of our experimental procedures is given in Fig. 1.

### BAC sequencing

#### Identification and analysis of gene-containing BACs

Isolation of gene-containing BACs, construction of a minimal tiling path (MTP), sequencing of MTP clones and the annotation of genes were essentially as described previously^13^.

#### Shotgun and mate-pair sequencing of MTP-BACs

Sequencing of MTP-BACs was conducted in four laboratories (Leibniz Institute on Aging—Fritz Lipmann Institute (FLI) Jena, Leibniz Institute of Plant Genetics and Crop Plant Research (IPK) Gatersleben, Beijing Genomics Institute (BGI) and Earlham Institute (EI) Norwich). Depending on the instrumentation and established protocols, customized approaches were taken to sequence the barley MTP BACs.

Barley chromosomes 1H, 3H and 4H (IPK and FLI)

*Shotgun sequencing of MTP BACs*

During the initial phase, BACs mostly from chromosome 3H (4870 clones) and a small number of clones from other chromosomes (34 from 1H; 31 from 2H; 50 from 4H; 101 from 5H; 33 from 6H; 64 from 7H; 107 from ‘0H’) were shotgun sequenced using the Roche/454 GS FLX device (Data Citation 1, Data Citation 2, Data Citation 3, Data Citation 4, Data Citation 5, Data Citation 6, Data Citation 7, Data Citation 8, Data Citation 9). BAC DNA was prepared using a modified alkaline lysis protocol^14^. Construction of barcoded 454 sequencing libraries and sequencing using the Roche platform were performed as described^15,16^. The remaining BAC clones from chromosomes 1H, 3H and 4H were shotgun sequenced employing Illumina instruments. BAC DNA isolation, library construction, sequencing-by-synthesis (paired-end, 2×100 cycles) using the Illumina HiSeq2000 device was performed as described^17^ (Data Citation 10, Data Citation 11, Data Citation 12, Data Citation 13). Pools of up to 667 BACs were individually barcoded and sequenced on one HiSeq2000 lane.

In addition, the Illumina GAIIx, HiSeq2500 and MiSeq machines were utilized to sequence pools of up to 384 clones per lane as described previously^17^.

*Mate-pair sequencing of MTP BACs*

For scaffolding of chromosomes 1H, 3H and 4H standard Illumina Nextera mate-pair libraries (span size: 8 kb) of BAC pools up to 384 BACs were constructed and sequenced using the Illumina HiSeq2000 (paired end, 2×100 cycles) and MiSeq (paired end, 2×250 cycles) as described^17^ (Data Citation 14, Data Citation 15).

Barley chromosomes 5H, 6H and 7H (BGI)

*Shotgun sequencing of MTP BACs*

Bacterial starter cultures were inoculated in 0.4 ml 2×YT liquid medium^18^ supplemented with chloramphenicol (17.5 μg ml^−1^) in 2 ml polypropylene 96-deep well-plates sealed with gas-permeable foil and incubated at 37 °C for 14 h in a shaking incubator (210 r.p.m.). For DNA isolation duplicates of cultures (1 ml 2×YT liquid medium containing 17.5 μg ml^−1^ chloramphenicol) were inoculated with 50 μl starter culture and incubated (37 °C, 14 h, 210 r.p.m.). BAC DNA was isolated using the alkaline lysis method essentially as described previously^17^. The DNA was dissolved (overnight, 4 °C) in 64 μl TE (pH 8.0) containing RNase A (30 μg ml^−1^) and stored at −20 °C. BAC plasmid DNA (0.5–2.0 μg in 60 μl) was randomly fragmented by focused-ultrasonicator (Covaris LE220 instrument: 21% duty factor, 500 PIP, 500 cycles per burst, 70 s treatment time) in 96-well plates (Axygen, PCR-96M2-HS-C) to an average size of 250–750 bp. The DNA fragments were purified using magnetic beads (GeneOn Purification kit, GO-PCRC-5000) according to the manufacturer’s instructions. DNA was precipitated by adding 10 μl magnetic bead suspension and 75 μl Binding Buffer. The samples were mixed and incubated at room temperature for 5 min. Beads containing the DNA were reclaimed by using a magnet (96S Super Magnet Plate, ALPAQUA, A001322), and the clear supernatant was discarded. The beads were washed twice with 200 μl of 70% ethanol and dried completely. For the elution of DNA the beads were suspended in 42 μl Elution Buffer (EB, 10 mM Tris-Cl, pH 8.5) and incubated (5 min). The plate was placed on the magnet, and the supernatant (40 μl) was transferred into new 96-well plates. End-repair and A-Tailing were performed as described^19^. The reaction clean-ups were performed with GeneOn magnetic beads as described above. Barcode adapters (1 μl, 20 μM) for the first index were ligated to the sticky ends of DNA fragments by using T4 DNA ligase^19^, incubated at 16 °C for at least 12 h. Each individual sample was provided with a different barcode of a set of 384 different indices (adapter and barcode sequences are available upon request). Equal volumes of the 384 individually barcoded adapter-ligated products were pooled. The pooled DNA was precipitated by adding 20 μl GeneOn magnetic beads and 650 μl Binding Buffer (GeneOn Purification Kit, GO-PCRC-5000) to 500 μl pooled DNA. The suspension was mixed and incubated at room temperature for 5 min. The beads containing the DNA were reclaimed using a magnet, and the clear supernatant was discarded. The beads were washed twice with 500 μl of 70% ethanol and dried completely. The DNA was eluted in 52 μl EB. The sample was size-separated by using standard agarose gel electrophoresis (2% agarose gel, HyAgarose, 16250). DNA was revealed using ethidium bromide and excitation by visible blue light emitted from a Dark Reader blue light transilluminator (Clare Chemical Research) to select the target fragments (580–620 bp). The target region was extracted in 27 μl EB using the QIAquick Gel Extraction kit (QIAGEN). The second index was introduced using the adapter-ligated products as template DNA (98 °C for 30 s, 10 cycles of: 98 °C for 10 s, 65 °C for 30 s and 72 °C for 30 s, final extension 72 °C for 5 min) (Enzymatics, CM0075) and PCR products (target region: 580–620 bp) were recovered by agarose gel electrophoresis (2% agarose gel, HyAgarose, 16250) as described above. Index primers were used for barcoding each 384 pooled BAC samples (index primer sequences are available upon request). The average size of the PCR products was determined by using an Agilent 2100 Bioanalyzer (Agilent DNA 1,000 Reagents). Typical average size of the libraries was between 574 to 674 bp. PCR products were quantified using real-time PCR and pooled for sequencing in equal proportion^20^. Paired-end sequencing (2×100 cycles; first index: 11 cycles, second index: 8 cycles) was performed on the Illumina HiSeq2000 platform (Data Citation 16, Data Citation 17, Data Citation 18).

*Mate-pair sequencing of MTP BACs*

For the construction of mate-pair libraries (10 and 20 kb span size), 96 BACs corresponding to 6 μg DNA were pooled into one tube. The DNA was fragmented to 10 or 20 kb by using the HydroShear DNA Shearing system from GeneMachines (10 kb: large assembly, speed code 12, cycles 12, volume 250 μl; 20 kb: large assembly, speed code 13, cycles 20, volume 150 μl). Following DNA fragmentation, the fragments were purified by using 0.6 volumes magnetic beads (Axygen, MAG-PCR-CL-250). The samples were mixed and incubated at room temperature for 10 min. Beads containing the DNA were reclaimed by using a magnet plate (96S Super Magnet Plate, ALPAQUA, A001322), and the clear supernatant was discarded. The beads were washed twice with 500 μl of 70% ethanol and dried completely. For the elution of DNA the beads were resuspended in 80 μl EB. End-repair and biotin-labeling were performed as described^21^. End-repaired DNA was purified using 0.6 volumes magnetic beads (Axygen, MAG-PCR-CL-250) as described for the purification of hydro-sheared DNA. The DNA was eluted in 79 μl EB. 20 kb libraries (20–26 kb range) were size-selected using agarose gel (0.6%) electrophoresis. The ligation of the libraries, was performed by adding 1 μl Barcode Adaptor (20 μM, sequences are available upon request), 10 μl T4 DNA ligase (Enzymatics, L603-HC) in a total volume of 100 μl (20 °C, 15 min). 15 individually barcoded adaptor-ligated DNAs (10 kb) were pooled in equimolar manner and size-fractionated (9–11 kb) using agarose gel (0.6%) electrophoresis. DNA circularization and removal of non-circularized DNA was as described^21^. The DNA was isolated from the gel using the QIAquick Gel Extraction kit as described by the manufacturer (QIAGEN). Circular DNA was fragmented using the Covaris S2 device (10% duty cycle, 10 intensity, 1,000 bursts per second, 22 min (11 min) treatment time for 10 kb (20 kb) libraries in TC13 Covaris tubes), and biotinylated fragments derived from true mate-pair ligation events were purified using streptavidin-coupled Dynabeads (M-280, Invitrogen)^19^. Ends of the DNA fragments were repaired and provided with Illumina paired-end adapters as described for the construction of shotgun libraries. The bead-bound DNA was PCR-amplified using Phusion polymerase (NEB) (98 °C for 30 s, 18 cycles of: 98 °C for 10 s, 65 °C for 30 s, 72 °C for 30 s and a final extension: 72 °C for 5 min) using manufacturer’s protocols (NEB). Size-selection was essentially performed as described for shotgun library construction. For the 10 kb (20 kb) mate-pair libraries, DNA in the size range between 270–420 bp (400–600 bp) was isolated and purified using the QIAquick Gel Extraction kit according to manufacturer’s instructions (QIAGEN). The average size of the paired-end BAC libraries was determined electrophoretically using an Agilent 2100 Bioanalyzer (Agilent DNA 1,000 Reagents). Libraries were quantified using Real-Time PCR^20^. The mate-pair libraries were paired-end sequenced using the Illumina HiSeq2500 device (10 kb library: 150 cycles, 20 kb mate-pair library 50 cycles). Raw data are available as Data Citation 19, Data Citation 20, Data Citation 21).

Barley chromosomes 2H and 0H (EI)

*Shotgun sequencing of MTP BACs*

QRep 384 Pin Replicators (Molecular Devices, New Molton, UK) were used to inoculate clones from stock plates into 384 square deep well culture plates containing 140 μl 2×YT media supplemented with 12.5 μg ml^−1^ chloramphenicol^18^. The culture plates were sealed with a gas permeable seal and incubated for 22 h at 37 °C in a shaking incubator (200 r.p.m.). Cells were harvested by centrifugation (20 min, 3,220 g, 4 °C), the supernatant was discarded. BAC DNA was prepared using a modified alkaline lysis protocol (Beckman Coultier, High Wycombe, UK). Cell pellets were resuspended in 8 μl of Resuspension Buffer (RE1) using a Microplate Shaker TiMix 5 control (Edmund-Buehler, Hechingen, Germany) (10 min, 1,400 r.p.m.). Cells were lysed by adding 8 μl of the lysis solution (L2). After shaking (5 min, 500 r.p.m.) 8 μl of cold Neutralisation Buffer (N3) were added. The plate was shaken (10 min, 500 r.p.m.) followed by a centrifugation (20 min, 3,220 g, 4 °C). The clear supernatant (14.33 μl) was transferred to a 384 well PCR plate, which contained 1 μl of CosMc beads per well. The plate was mixed briefly (500 r.p.m.), 10 μl of isopropanol was added and the suspension was mixed briefly again (500 r.p.m.). The plate was incubated at room temperature for 15 min to allow precipitation of the DNA onto the beads. The plate containing the DNA precipitate was moved onto a 96 pin 384 well plate compatible magnet (Alpaqua, Beverley, MA, USA) and left for 5 min for the beads to pellet. The supernatant was discarded and the beads were washed three times with 20 μl 70% ethanol while placed in the magnet and air dried (room temperature, 5 min). The DNA was eluted from the beads in 20 μl of 10 mM Tris HCl (pH 8.0) and transferred to a fresh 384 well PCR plate. To remove contaminating host E. coli gDNA samples were treated with Epicentre Plasmid Safe ATP dependent DNase (Cambio, Cambridge, UK), which digests the fragmented E. coli and nicked BAC DNA but leaves supercoiled BAC DNA intact. To 20 μl of DNA 2.5 μl of 10x Reaction buffer, 1 μl 25 mM ATP, 0.1 μl ATP dependent DNase (10 u μl^−1^) and 1.4 μl water was added, and the samples were incubated at 37 °C (8 h) followed by 70 °C (20 min) to inactivate the DNase. Sequencing libraries (single index) from the initial sixteen 384 well plates of BACs (2H chromosome) were constructed in 384 well PCR plates (Fortitude, Wotton, UK) using the Epicentre Nextera Kit (Epicentre, Madison, WI, USA) and Robust 2G Taq polymerase (Kapa Biosciences, London, UK). The 384 adapter oligos with 9 bp barcodes each with a hamming distance of 4 (adapter sequences are available upon request) were designed using standard guidelines^22^. Briefly, 1 μl of BAC DNA, 1 μl Nextera HMW 5×Reaction Buffer, 1 μl of Nextera Enzyme (diluted 50-fold in 50% glycerol, 0.5×TE pH 8.0) and 2 μl of water were combined and incubated (5 min, 55 °C) as described^23^. For the denaturation of the Tn5 polymerase, 15 μl PB Buffer (Qiagen, Manchester, UK) and for the reaction clean-up, 20 μl AMPure XP (Beckman, High Wycombe, UK) beads were added using a Caliper Sciclone Robot (Perkin Elmer, Coventry, UK). Following an incubation (5 min, room temperature), the precipitated tagmented DNA was purified using a 96 well ring Magnet (Alpaqua, Beverly, MA, USA). The beads were washed twice with 20 μl 70% ethanol while placed in the magnet before being air dried for 5 min. The tagmented DNA was eluted in 5 μl 10 mM Tris HCl, pH 8.0 and transferred to a fresh 384 well PCR plate. To 5 μl purified, tagmented DNA 2 μl of 5×2G B Reaction buffer, 0.2 μl of 10 mM dNTPs, 0.1 μl of Robust 2G Taq polymerase, 0.2 μl of 50×Nextera Primer Cocktail and 2.5 μl 0.2 μM barcoded P2 adapter primer were added in a total reaction volume of 10 μl and amplified according to the following thermal cycling profile: 72 °C for 3 min, 95 °C for 1 min, followed by 21 cycles of 95 °C for 10 s, 65 °C for 20 s and 72 °C for 3 min. Post amplification the DNA concentration was determined using the Quant-It Picogreen dsDNA assay (Thermo Fisher, Cambridge, UK). Library DNA concentrations typically ranged from 4 to 40 ng μl^−1^ (average of 16 ng μl^−1^). For each sample from a 384 well plate a 5 μl aliquot was pooled and split into two 2 ml Lo bind Eppendorf tubes (950 μl each). To each aliquot 950 μl of AMPure XP (Beckman, High Wycombe, UK) beads was added. Samples were mixed, incubated (5 min, room temperature) and placed on a magnet particle concentrator (MPC) until the beads were collected. The supernatant was discarded. The beads were washed twice with 20 μl 70% ethanol while placed in the MPC and air dried (5 min). The pooled library was eluted from the beads in 17 μl of 10 mM Tris HCl pH 8.0. The two 17 μl aliquots of the library were combined and the DNA concentration was determined using the Qbit device with the Quant-It DNA HS Assay (Invitrogen). Typical DNA concentrations were above 100 ng μl^−1^. The DNA size selection was performed using the Blue Pippin (Sage Science, Beverly, MA, USA). About 3 μg of the library in 30 μl of 10 mM Tris HCl pH 8.0 and 10 μl of the R2 ladder were separated (tight selection protocol, 650 bp) using a 1.5% agarose cassette according to the manufacturer’s instructions (Sage Science, Beverly, MA, USA), thereby yielding an average insert size of about 485 bp. Size selected samples were collected in 40 μl of TRIS- TAPS buffer, pH 8.0 (Sage Science, Beverly, MA, USA). The average size of the library was determined using a High Sensitivity Chip and an Agilent 2100 Electrophoresis Bioanalyzer (Agilent). The DNA concentration was measured using the Qbit device and the Quant-It DNA HS Assay (Invitrogen). Size selected libraries were quantified using the Kappa Biosciences Illumina library qPCR quantification kit (Kapa Biosciences) on a Step One qPCR machine (ThermoFisher) according to the manufacturer’s instructions and compared against a known concentration of a PhiX control library. Several libraries were pooled for sequencing in an equimolar manner, and the final pool was re-quantified for sequencing relative to a standard library of a known concentration using the Kapa Biosciences Illumina library qPCR quantification kit. Sequencing-by-synthesis for 6,144 BACs from chromosome 2H was performed using an Illumina HiSeq2000 device (2×100 cycles paired-end, single indexing read, 384 BACs/lane) according to manufacturer’s instructions, thereby yielding at least 32 Gb/lane and an average sequence coverage of at least 500-fold per BAC. The remaining BAC clones from 2H (384 BACs/lane) and 0H (2304 BACs/lane) were sequenced with a HiSeq2500 machine (2×150 cycles paired-end, dual indexing, rapid mode, yield: at least 30 Gb/lane) using a slightly adapted protocol with an additional normalization step prior to sample pooling. Briefly, a custom panel of 48 P5 and 48 P7 adapter oligos with 9 bp barcodes (with ≥4 hamming distance) was designed to individually label up to 2,304 (48×48) libraries by dual indexing. A mixture of 2 μl of BAC DNA, 0.5 μl Nextera 10×Reaction Buffer, 0.1 μl Nextera Enzyme and 2.4 μl water was incubated (5 min, 55 °C). Tn5 denaturation, reaction clean-up, washing, elution and transfer to a fresh 384 well plate were as described for the single-indexing libraries. 5 μl purified, tagmented DNA, 2 μl of 5×Kapa Robust 2G B Reaction buffer, 0.2 μl of 10 mM dNTPs, 0.05 μl of Kapa Robust 2G Taq polymerase, 1 μl 2 μM P5 primer, 1 μl 2 μM P7 primer were combined (reaction volume of 10 μl) and amplified according to following thermal cycling profile: 72 °C for 3 min, 95 °C for 1 min, followed by 16 cycles of 95 °C for 10 s, 65 °C for 20 s and 72 °C for 3 min. The size profile and quantity was determined as described for single-indexing libraries. Amplified libraries were normalised using MagQuant bead technology (GC Biotech, Netherlands) on a Caliper Zephyr Robot (Perkin Elmer), essentially as described by the manufacturer. Normalised libraries were eluted in 10 μl of 10 mM Tris HCl pH 8.0 and transferred to a fresh 384 well PCR plate.5 μl of 384 normalized samples were pooled (total volume 1,920 μl). Purification using AMPure XP beads, washing, elution, size-selection (Blue Pippin) and quality checks prior to sequencing were essentially as described for single indexing libraries. Sequencing-by-synthesis of pooled libraries (2,304 BACs) was performed using an Illumina HiSeq2500 device (rapid run mode, 2×150 cycles paired-end, dual indexing reads) according to manufacturer’s instructions. At least 40 Gbp/lane, and an average sequence coverage of >100-fold per BAC were obtained (Data Citation 22, Data Citation 23, Data Citation 24, Data Citation 25).

*Mate-pair sequencing of MTP BACs*

BAC clones were inoculated as described for the preparation of shotgun libraries. The bacterial cultures were grown for 6 h at 37 °C in a shaking incubator at 200 r.p.m., and 384 clones were pooled. The pool was used to inoculate 250 ml 2×YT media supplemented with chloramphenicol (12.5 μg ml^−1^). The cultures were incubated (18 h, 37 °C, 200 r.p.m.). Cells were harvested by centrifugation (3,220 g, 20 min, 4 °C), and the supernatant was discarded. Alkali lysis and DNA isolation steps were performed using the Large Construct kit (Qiagen, UK) essentially following the manufacturer’s instructions. The DNA was resuspended in 4.75 ml Buffer Ex, 100 μl 100 mM ATP (Fisher Scientific, UK) were added and contaminating E. coli DNA was removed using 150 μl ATP dependent Exonuclease (Qiagen). During the incubation (1 h, 37 °C) a Qiagen Tip-100 column (Qiagen) was equilibrated in Buffer QBT (Qiagen). 5 ml of Buffer QS were added to the DNA, and the sample was applied to the equilibrated column. The column was washed twice with 10 ml of Buffer QC (Qiagen). The DNA was eluted with 7.5 ml of pre-warmed (65 °C) Buffer QF (Qiagen). The DNA was precipitated by adding 0.7×volume of room temperature isopropanol and centrifugation (20 min, 3,220 g, 4 °C). The pellet was washed twice with 70% ethanol, air dried and dissolved in 200 μl TE buffer according to manufacturer’s guidelines. The DNA concentration was measured using a Qubit Fluorometer (Thermo Fisher, Cambridge, UK) and adjusted with water to 13 ng μl^−1^. For tagmentation 200 μl diluted DNA were equilibrated (6 min, 55 °C) and subsequently provided with 52 μl 5×Tagment Buffer Mate-Pair and 8 μl Mate-Pair Tagmentation Enzyme (Illumina, San Diego, USA). After the incubation (30 min, 55 °C), 65 μl Neutralize Tagment Buffer (Illumina, San Diego, USA) were added, and the reaction was incubated (5 min, room temperature). One volume CleanPCR beads (GC Biotech, Alphen aan den Rijn, The Netherlands) was added, and the DNA was purified using magnetic separation. The DNA was eluted in 170 μl of nuclease-free water, quantified using a Qubit fluorometer (DNA HS assay, Invitrogen) and analysed using the Agilent Bioanalyser (DNA 1,200 chip, Agilent, Stockport, UK). Strand displacement was performed by combining 105.3 μl of tagmented DNA, 13 μl 10x Strand Displacement Buffer (Illumina), 5.2 μl dNTPs (Illumina), 6.5 μl Strand Displacement Polymerase (Illumina) and incubation (30 min, room temperature). CleanPCR beads (0.75 volume) were added and the DNA was purified using a magnet. The DNA was eluted in 30 μl nuclease-free water. The concentration was measured (Qubit, DNA HS assay, Invitrogen), and a 1:6 diluted sample was analysed using the Agilent Bioanalyser (DNA 1,200 chip, Agilent, Stockport, UK). Size selection was performed using a Pippin Blue (Sage Science, Beverly, MA, USA). 30 μl DNA were provided with 10 μl loading buffer and separated on a 0.75% agarose cassette (size selection centered at 7 kb and collection between 6–8 kb) according to the manufacturer’s instructions (Sage Science, Beverly, MA, USA). Size selected samples were collected in 40 μl of TRIS- TAPS buffer (pH 8.0) (Sage Science, Beverly, MA, USA), and analysed using the Agilent Bioanalyser (high sensitivity chip, Agilent, Stockport, UK) to determine the final library size. The DNA concentration was measured using the Qubit device and the Quant-It DNA HS Assay (Invitrogen). Circularisation was performed by combining 40 μl size selected DNA, 12.5 μl 10×circularisation buffer (Illumina), 3 μl Circularisation Enzyme (Illumina) and 75 μl nuclease-free water. The reaction was incubated at 30 °C overnight. Linear DNA was digested by adding 3.75 μl Exonuclease (Illumina) and incubation (30 min, 37 °C). The enzyme was inactivated by heat (30 min, 70 °C) and the addition of 5 μl stop ligation (Illumina). Circularised DNA (130 μl) was sheared in a Covaris MicroTube AFA Fiber (Pre-slit, Snap-cap, 6×16 mm; 2 cycles of 37 s, 10% duty cycle, 200 cycles per burst, 4 intensity, 4 °C) using the Covaris S2 device (Covaris, Massachusetts, USA). M280 Dynabeads (Thermo Fisher) were prepared as described (Illumina). 130 μl washed M280 beads were added to the fragmented DNA, mixed and placed on a lab rotator (20 min, room temperature). Library molecules were affinity purified and washed as described (Illumina). The beads were resuspended in a mixture of 85 μl nuclease free water, 10 μl 10x End Repair Reaction Buffer (Ilumina) and 5 μl end repair enzyme mix (Illumina) and incubated (30 min, 30 °C). End repaired library molecules bound to M280 beads were washed as described (Illumina). A-Tailing and adapter ligation were performed according to manufacturer’s instructions (Illumina). For PCR amplification, the beads were resuspended in a reaction mixture (20 μl nuclease-free water, 25 μl 2x Kappa HiFi (Kappa Biosystems, London, UK), 5 μl Illumina Primer Cocktail) and amplified (98 °C for 3 min, 12 cycles of 98 °C for 10 s, 60 °C for 30 s, 72 °C for 30 s followed by 72 °C for 5 min and storage of the sample at 4 °C). Beads were removed by magnetic separation and 45 μl of the products were transferred to a 2 ml DNA Lobind Eppendorf tube. The DNA was precipitated by addition of 31.5 μl CleanPCR beads (GC Biotech, Alphen aan den Rijn, The Netherlands). The beads were washed twice with 100 μl 70% ethanol, and the final library was eluted in 20 μl resuspension buffer (GC biotech). The DNA concentration was determined (Qubit, DNA HS assay, Invitrogen), followed by analysis using the Agilent Bioanalyser (High sensitivity chip, Agilent, Stockport, UK). Up to 12 mate-pair libraries were pooled in an equimolar manner and measured using the Kappa qPCR Illumina quantification kit. Sequencing-by-synthesis of pooled mate-pair libraries was performed using an Illumina HiSeq2500 device (rapid run mode, 2×150 cycles paired-end, single indexing reads) according to manufacturer’s instructions (Data Citation 26, Data Citation 27).

### Sequence assembly of individual BACs

#### Assembly of gene-containing BACs (UCR/JGI)

A total of 15,661 gene-bearing BACs were paired-end sequenced (2×100 cycles) using the Illumina HiSeq2000 platform (Illumina, Inc., San Diego, CA, USA) applying a combinatorial pooling design^24^, as described in Munoz-Amatriain *et al.*^13^. Reads were quality trimmed, deconvoluted, and then assembled BAC-by-BAC using Velvet version 1.2.09 (ref. 25) with the parameter k set to 45. Sequences of an additional 50 randomly chosen BACs included in Munoz-Amatriain *et al.*^13^ were derived using the Sanger method by Jane Grimwood (US Department of Energy Joint Genome Institute) and Jeremy Schmutz (HudsonAlpha Institute for Biotechnology), including shatter and transposon sequencing. The assignment of BACs to chromosome arms/peri-centromeric regions was performed using CLARK^26^, an accurate *k*-mer-based classification method that is much faster than BLASTN or MegaBLAST. CLARK makes assignments by using a prebuilt database of *k*-mers that are specific to each chromosome arm/peri-centromeric region.

#### Assembly of MTP BACs from barley chromosomes 1H, 3H, 4H, 6H and 7H (FLI and IPK)

A total of 10,148 BACs mainly originating from barley chromosome 3H were sequenced on the Roche 454 system. Reads were deconvoluted and assigned to individual BACs^16^. Reads were quality trimmed according to the manufacturer’s recommendations. Reads were screened for *E. coli* and vector sequences with MegaBLAST^27^. Assemblies were then constructed from the clean reads using the MIRA software^28^ as described in Steuernagel, *et al.*^16^ and Taudien, *et al.*^29^.

A total of 41,004 BACs were sequenced on Illumina machines (mainly HiSeq2000) in pools of up to 672 individually barcoded BAC clones. Paired-end reads were quality trimmed with the CLC toolkit and screened for *E. coli* and vector sequences with MegaBLAST. Assemblies were obtained by running CLC Assembly Cell Version 4.0.6 beta with default parameters. Contigs derived with low read coverage as well as contigs smaller than 500 bp were removed using the criteria described in Beier, *et al.*^17^.

The resultant contigs were then compared to NCBI’s nucleotide database using MegaBLAST to check for possible contamination. Contigs with non-plant hits were either completely removed or trimmed.

#### Scaffolding of MTP BACs from barley chromosomes 1H, 3H, 4H, 6H and 7H (FLI and IPK)

Scaffolding was performed as described in Beier *et al.*^17^ Briefly, mate-pair reads were mapped against the concatenated assemblies of up to 384 BACs using BWA mem version 0.7.4 (ref. 30) with default parameters. Only read pairs mapping uniquely (minimal mapping quality of Q40) to different contigs of the same BAC assembly were retained. These reads were used to scaffold individual BACs using SSPACE version 3.0 Standard^31^.

If multiple mate-pair libraries were present (MiSeq mate-pair reads as well as HiSeq2000 mate-pair reads) an iterative scaffolding procedure^17^ was used.

#### Assembly of MTP BACs from barley chromosome 5H (BGI)

Obtained raw sequence reads from 5H MTP BACs were filtered to generate high-quality reads by the following criteria: (1) reads containing more than 2% of Ns or with poly-A structures were removed; (2) reads with≥40% low quality bases for short insert size libraries (60% for large insert size libraries) were excluded; (3) reads containing adapters were removed; (4) PCR duplicates were detected and excluded; (5) removal of reads contaminated by *E. coli*, vector sequences or phage sequences. High-quality reads were then used for assembly.

BACs were assembled using SOAPdenovo version 2.01 (ref. 32) multiple times using different k and m values (main parameter in SOAPdenovo assembly). In total each BAC was assembled 45 times (k from 33 to 66, only odd numbers and m from 1 to 3). The N50 was examined for each assembly and the assembly with the largest N50 was retained as the final assembly result for each BAC.

#### Scaffolding of MTP BACs from barley chromosomes 5H (BGI)

Assemblies from paired-end sequences were used as reference for mapping 2, 5 and 10 kb mate-pair reads obtained from barley genomic WGS data with SOAPaligner/soap2 version 2.21 with parameters –p 6 –v 3 –R. Mate-pair read pairs mapped in this fashion were used in conjunction with the corresponding paired-end read pairs to re-assemble each BAC using SOAPdenovo version 2.01 as described above.

#### Assembly of MTP BACs from barley chromosomes 2H and ‘0H’ (EI)

Minimal tiling path BACs from (i) barley chromosomes 2H or from (ii) fingerprinted contigs not assigned to chromosomes (termed ‘0H’) were sequenced. After demultiplexing, sample quality control (QC) information was generated using FastQC^33^. Contamination screening was carried out using Kontaminant^34^. Reads were screened using a *k*-mer size of 21 against a range of potential contaminants (Phi X, *E. coli*, *Enterobacter cloacae* genomic DNA and BAC vector) and contaminated reads or reads with quality values <30 were removed.

ABySS assembler (v1.5.1)^35^ was used to assemble the filtered paired-end reads of each BAC individually (*k*-71, l-91 b-0). Paired-end contigs were compared to NCBI’s NR database using BLAST to check for hits to non-plant organisms using e-value 1e-4 as threshold. The obtained hits were compared to NCBI taxonomy using ‘fastacmd’ to obtain common names used to check for any non-plant hits.

#### Scaffolding of MTP BACs from barley chromosomes 2H and ‘0H’ (EI)

Illumina Nextera mate-pair libraries were created from pools of 384 BACs. After quality checking the reads using PAP^34^, the reads were merged using FLASH (version 1.2.9)^36^. Nextclip (v0.8)^37^ was run on the flashed reads to trim the junction adapters. A *k*-mer-based approach was used to assign mate-pair reads to individual BACs with KAT (v1.0.4) (https://github.com/TGAC/KAT). Scaffolding and gap closing were performed on each BAC individually using an in-house shell script (available from GitHub: https://github.com/DhSaTGAC/BAC-assembly-pipeline.git). SOAPdenovo scaffolder version 2.01 (ref. 38) was applied to scaffold the ABySS paired-end contigs using the *k*-mer classified mate-pair reads with parameters *k*=41, -G 30, -F, -w and -L 100. The resulting scaffolds were then edited to replace long stretches (>20) of C/G with ‘N’ characters as SOAP is known to substitute ‘N’s within paired-end contigs to C/G. The scaffolds were then passed through GapCloser (v1.12-r6), a SOAP2 module, to fill in long stretches of ‘N’s produced during the scaffolding steps. Contigs and scaffolds shorter than 500 bp were removed to produce the final assembly per BAC.

#### Splash contamination checks of MTP BACs from barley chromosomes 2H and ‘0H’ (EI)

The raw reads within each plate were aligned to one side of the vector sequence adjacent to the restriction enzyme cut site using exonerate^39^. Substrings of size 20 bp were extracted from aligning reads containing the BAC sequence adjacent to the vector sequence. Flanking sequences from each BAC were clustered based on a Hamming distance<3 and consensus sequences generated to account for sequencing errors. These were compared with neighboring wells to check for potential contamination caused by splash during lab processing steps. Where contamination between neighboring wells was indicated, the assembled contigs from each BAC in question were aligned in a pairwise fashion using exonerate and the total percentage of similar sequence (≥ 99% identity) was computed. In cases where neighboring BACs shared more than 10% similar sequence, both BACs were resequenced.

### Pseudomolecule construction

#### Initial contamination removal

Sequence assemblies of 66,586 MTP clones, 5,468 non-MTP BACs and 15,044 gene-bearing clones^13^ (total number of unique BACs: 87,098) were combined into a single FASTA file (Data Citation 28,Data Citation 29,Data Citation 30). If a clone had two or more independent sequence assemblies, we selected the one with the largest N50 value for further analyses. BAC assemblies were aligned to a custom library of potential contaminants (Data Citation 31) including phages, bacterial and vector sequences using megablast^27^. Regions aligning to contaminants (criteria: (alignment length≥500 bp **AND** identity≥80%) **OR** (identity≥90%)) were removed from the assembly using UNIX scripts and BEDTools^40^. Sequences shorter than 500 bp or consisting of less than 500 proper nucleotides (ACGT characters) after contamination removal were discarded. This step removed 55.5 Mb (0.5%) of the assembled BAC sequence.

#### Sequence alignment of BACs sequences and overlap detection

After contamination removal, a set of 87,075 BAC assemblies (Table 1, Data Citation 32) was aligned against itself using megablast^27^ with a word size of 44, retaining only alignments with identity≥99% and alignment length≥500 bp. Two sets of overlaps (stringent and permissive) between BACs were defined from the BLAST results of all BACs against each other. Pairs of BACs were considered as potentially overlapping under stringent criteria if there was at least one high-scoring pair (HSP) with alignment length≥5 kb and identity≥99.8%. Under permissive criteria, we required at least one HSP with alignment length≥2 kb and identity≥99.5%. For all pairs of potentially overlapping BACs (under either set of criteria), the size of their overlapping regions was determined using UNIX scripts and BEDTools^40^ as the extent of non-redundant regions in the BAC sequences (i.e., contigs or scaffolds) contained in HSPs≥500 bp and identity≥99.5% between BAC sequences having at least one HSP with alignment length≥5 kb and identity≥99.8% (stringent criteria) or alignment length≥2 kb and identity≥99.5% (permissive criteria). HSPs less than 200 bp apart were combined into one with BEDTools (command ‘merge’). BAC overlap information was imported into the R statistical environment^41^ for use in genetic anchoring and merging sequence assemblies of adjacent BAC clones (see section ‘Construction of the BAC overlap graph’).

#### Alignment of BACs to the BioNano map of barley cv. Morex

An optical map of the genome of barley cv. Morex was generated using the Irys platform of BioNano Genomics using Nt.*Bsp*QI as the nicking enzyme. Further details of the optical map procedure are described in Mascher *et al.*^42^ An *in silico Bsp*QI digest was performed with the Knickers software (http://www.bionanogenomics.com) using default parameters. Restriction maps of BAC sequences were aligned to the BioNano map of barley cv. Morex^42^ (Data Citation 33) with IrysView software^43^ (http://www.bionanogenomics.com) using the command line tool RefAligner (version 3827) with the following parameters ‘-M 2 -T 1e-4 -extend 1 -biaswt 0’ to report all alignments with a confidence score≥4.

#### Construction of the updated POPSEQ map of the Morex x Barke mapping population

An ultra-dense linkage map had been constructed previously^5^ by shallow whole-genome shotgun sequencing of 90 recombinant inbred lines (RILs) derived from a cross between the barley cultivars Morex and Barke. We wished to increase the resolution of this map by reducing the average fraction of missing data per SNP marker. Towards this aim, we sequenced the existing Illumina paired-end libraries of 87 RILs to higher coverage (2–3x) and combined them (Data Citation 34) with the existing read data set^5^ (ENA accession: ERP002184). Map construction followed the procedures described in Chapman *et al.*^44^. Reads were aligned to the whole-genome shotgun assembly of barley cv. Morex^4^ (NCBI accession: CAJW01) with BWA mem version 0.7.5a (ref. 45). Sorting, conversion to BAM format and removal of duplicate reads was done with PicardTools version 1.100 (http://broadinstitute.github.io/picard/). Variant detection and genotype calling were performed with SAMTools version 0.1.19 (commands ‘samtools mpileup –BD’ and ‘bcftools view –cvg’). The resultant VCF file was filtered using an AWK script (Supplementary Text S3 of Mascher *et al.* 2013 (ref. 46)). Homozygous genotype calls were set to missing if their read depth was 0 or their genotype quality below 3. Heterozygous genotype calls were set to missing if their read depth was below 3 or their genotype quality below 5. Variants with (i) a quality scores below 40, (ii) more than 10% heterozygous genotype calls, (iii) more than 90% missing data after genotype call filtering, or (iv) a minor allele frequency below 5% were discarded. SNP information was aggregated at the contig level to derive consensus genotypes as described in the section ‘Framework map construction’ in the Methods section of Chapman *et al.*^44^ For map construction with MSTMap^47^, the population type ‘RIL8’ was used. Additional contigs were inserted into the framework map as described in Chapman *et al.*^44^ (section ‘Anchoring scaffolds onto the framework map’) using previously published read data^5^. Variant calling and map construction were done for the Oregon Wolfe Barley (OWB) doubled haploid population using the same procedures with the following two changes: (i) heterozygous genotype calls were excluded and (ii) the population type ‘DH’ was used for map construction with MSTMap^47^. Map positions in the OWB map were interpolated into the Morex x Barke map using loess regression in R^41^. A consensus position was derived as follows: if map positions disagreed by more than 5 cM in both maps, a contig was considered unanchored; otherwise, the Morex x Barke position was preferred if available. The final map assigned genetic positions to 791,176 WGS contigs (Table 2, Data Citation 35), compared to 723,499 anchored contigs in the original POPSEQ map^5^.

#### Genetic anchoring of single BAC clones

The genetic positions of Morex WGS contigs in the updated POPSEQ map were lifted to BAC sequences via sequence alignment. The set of all contigs of the whole-genome shotgun assembly of barley cv. Morex^4^ (NCBI accession: CAJW01) was aligned to all BAC assemblies with megablast^27^ using a word size of 44 and retaining only alignments with identity≥99.8% and alignment length≥1,000 bp. For each BAC clone, the genetic positions of WGS contigs aligning to its constituent sequences were tabulated and a genetic position of a clone was derived using a majority rule with functions of the R package ‘data.table’ (https://cran.r-project.org/web/packages/data.table/index.html). Ninety per cent of contigs assigned to a BAC had to originate to the major chromosome and the standard deviation of genetic positions had to be≤3 cM. BACs without alignments to anchored WGS contigs were considered as unanchored; those not meeting the consistency criteria were flagged as ‘inconsistently anchored’. In the second step, unanchored clones were positioned by utilizing positional information from neighboring BACs. We considered as neighbors of a given clone B all those BACs that overlapped for at least 10% of their assembled lengths with clone B. The genetic position of an unanchored BAC B with an assembled length≤300 kb were borrowed from its neighbors if all of them were anchored to same chromosome and the standard deviation of genetic coordinates was at most 3 cM. If these criteria were fulfilled, the genetic position of B was set to the arithmetic mean of the genetic coordinates of its neighbors. Genetic positions were determined for 78,693 (90.4%) BACs (Table 1, Data Citation 36).

#### Construction of the BAC overlap graph

We converted the overlap information between BACs in a graph structure using the R package ‘igraph’^48^. Nodes represented BACs. An edge was drawn between two nodes (BACs) if the criteria regarding sequence overlap and consistency of positional information were fulfilled as detailed below. The edge weights were set to the cumulative length of intervals in which two adjacent BACs overlapped. We named the connected components of this graph ‘clusters’. These clusters are analogous to physical contigs in that they represent overlaps between BACs. In contrast to physical contigs, overlaps between BACs in the cluster graph are not derived from restriction maps, but from sequence alignments.

The initial overlap graph was refined in subsequent steps by adding edges that were supported by (i) additional information about links between BACs derived from BAC end sequences, (ii) the genome-wide physical map of barley^3^ or (iii) the BioNano map. After each refinement step, we checked for the existence of branches in the overlap graph. Such branches should not occur in a linear genome and may have arisen from spurious sequence alignments or incorrect positional information. We also determined genetic locations of clusters by aggregating the positional information of their constituent BACs using a majority rule, requiring all anchored BACs to come from the same chromosome and the standard deviation of their genetic coordinates to be≤5 cM. Clusters not meeting these criteria were considered inconsistently anchored. Edges giving rise to branches or to inconsistent genetic positions were detected and removed. To detect branches, we calculated a minimum spanning tree (MST) of each cluster using Prim’s algorithm^49^ as implemented in the igraph^48^ function ‘minimum.spanning.tree()’. A geodesic of the MST of maximal length was determined with the igraph function ‘get.diameter()’ and set as the linear (i.e., branchless) backbone of the cluster. In the MST, each BAC B was either part of the diameter or attached to a single BAC of the backbone, i.e., there existed a path from B to one and only one BAC of the backbone. The length of this path to a member of the backbone was defined as its rank. Groups of BACs attached the same backbone BAC were considered as a ‘BAC bin’ of the cluster. Branches were defined as groups of nodes with rank>1. A cluster was said to be branched if it contained branches, i.e., had a non-linear structure. Note that due to redundancies in the BACs selected for sequencing, we expect BACs with rank equal to 1. After each insertion or removal of edges or nodes, connected components, MST backbones and genetic positions of clusters were re-calculated, and branches and inconsistencies with genetic data removed if necessary. The summary statistics of the overlap graph after each step are given in Table 3. The final clustering results summarized in Table 4 are available as Data Citation 36).

Step 1: Initial overlap graph from links within FP contigs

In the initial overlap graph, an edge between two BACs was drawn if both BACs were (i) on the same fingerprinted (FP) contig, (ii) the overlapping regions between them accounted for≥5% of the length of either BAC and (iiiA) there were genetically anchored to the same chromosome within 3 cM of each other or (iiiB) one or both clone were unanchored. To determine overlap lengths, we used the permissive set of overlaps. BACs that were inconsistently anchored or whose assembled length was>300 kb were excluded from the graph. The initial graph had both branched and inconsistently anchored clusters. To remove inconsistencies in genetic positions, all edges involving unanchored clones were deleted in clusters showing inconsistent genetic positions. To remove branches in the initial graph, we first removed nodes representing non-MTP clones that were part of branches. This step was iterated twice. In the next steps, BACs in branches and originating from the set of gene-bearing BACs^13^ were excluded. These BACs were sequenced using combinatorial pooling strategy and errors during demultiplexing may have given rise to chimeric assemblies. After these steps, nine clusters with branches remained in the graph. BACs in these branches were removed from the graph. After these steps, the graph was unbranched and showed no inconsistencies with the genetic map. The graph consisted of 9,637 clusters and 13,211 singletons (Table 3).

Step 2: Adding links between FP contigs

Next, we added edges between BACs on different FP contigs. An edge between two BACs was drawn if (i) the overlapping regions between them accounted for≥10% of the length of either BAC and (iii) they were genetically anchored to the same chromosome within 3 cM of each other. Stringent overlap criteria were used in this step. This graph had branches, which were removed in subsequent steps. First, clones shorter than 50 kb or having an N50<10 kb were excluded. Then, nodes representing non-MTP clones that were part of branches were deleted. This step was repeated once. Then, edges where both clones were part of branches and in different FPCs were removed, followed by another removal of non-MTP clones. In the next step, clones in branches that were longer than 250 kb were removed. These large assemblies may combine sequences of two unrelated BACs as a result of chimeric inserts or cross-contamination between neighboring well positions. Next, gene-bearing clones^13^ in branches were deleted. Finally, all remaining clones in branches were discarded. The resultant graph had no branches and all its clusters were consistently anchored to the genetic map. This step reduced the number of clusters from 9,637 to 4,980 and led to the exclusion of 1,166 putatively chimeric BAC assemblies giving rise to non-linear structures (Table 3).

Step 3: Adding links with permissive overlap criteria, but support by the BioNano map

In the next steps, we tried to find additional links between BACs that would support the joining of neighboring clusters. This was motivated by our desire to have fewer, but large clusters (i.e., increase the contiguity of the overlap graph) to facilitate the construction of the Hi-C map (see below). Towards this aim, we added edges to the graph using less stringent overlap criteria, but requiring support from other datasets. If the inclusion of an edge gave rise to a branch or map inconsistencies, this edge was removed again. We note that in some cases edges do not represent true sequence overlaps between BACs, but only evidence for close proximity of two BACs.

In the first step, we added edges between two BACs if (i) they were located at the ends of clusters, (ii) the overlapping regions between them accounted for≥10% of the length of either BAC, (iii) they were genetically anchored to the same chromosome within 3 cM of each other and (iv) and the link was supported by the BioNano map. The BACs at the ends of clusters were determined from the MST traversals of clusters. Support by the BioNano map means the presence of a single contig of the BioNano map (an ‘optical genome map’ (OM) in BioNano’s nomenclature) that links to two clusters. To find such genome maps, we aggregated the alignment information between BAC sequences and OMs at the level of clusters. In the alignment table between BioNano maps and BAC sequences, we only retained the best alignment of each BAC sequence contig. A cluster was considered aligned to a OM if the sum of the confidence scores (as reported by BioNano’s refaligner software) of its BAC sequences was at least 25. A OM was joining two clusters if (i) the distance in the OM between restriction map alignments pertaining to the two clusters was (i)≤300 kb and (ii) the order and orientation of alignments to the OM were consistent with the order of BACs in the MSTs of the clusters, requiring a rank correlation above 0.5. Adding all edges meeting these criteria to the overlap graph did not result in branches or inconsistent map positions within clusters. The graph consisted of 4,843 clusters (Table 3).

Step 4: Adding links supported by FP contigs, BAC end sequences and the BioNano map

We added edges representing pairs of BAC end sequences linking BACs at ends of clusters on the conditions that (i) these links were supported by the BioNano map and (ii) the joined BACs originated from the same FPC contig. BAC end sequences of cv. Morex (EMBL ENA accessions: HF140858-HF362636, HE975059-HE977519, HF000001-HF140857, HE867107-HE939654, HE939655-HE956691 and HF362637-HF479769) were aligned to all BAC assemblies with megablast^27^ using a word size of 28 and considering only hits with identity≥99.5% and alignment length≥500 bp. We identified pairs of BAC end sequences that aligned to BACs B1 and B2 from two different clusters C1 and C2. BACs B1 and B2 were required to be the end of their clusters and to belong to same FPC contig and were less than 200 kb apart from each other in the physical map (using the conversion factor 1 FPC consensus band=1.24 kb^3^) map. Moreover, we required the clusters C1 and C2 to be connected by a BioNano contig under the criteria described in the section ‘Adding links with permissive overlap criteria, but support by the BioNano map’. If all these criteria were fulfilled, we added an edge between B1 and B2. This step did not introduce branches or inconsistently anchored clusters to the graph. The number of clusters decreased to 4,653 (Table 3).

Step 5: Adding links supported by FP contigs and BAC end sequences

In this step, we used BAC end sequences and FP information to find additional links as described in the previous step, but we did not require support by the BioNano map. This step introduced branches to the graph that were removed by pruning newly introduced edges between BACs in branches. The updated graph was composed of 4,562 clusters (Table 3).

Step 6: Using FP information and inconsistently anchored BACs to bridge gaps

In previous steps, we had excluded inconsistently anchored BAC assemblies from the overlap analysis. We speculated that many of these assemblies may contain BAC sequences from two unlinked genomic loci as a consequence of chimeric inserts or cross-contamination between neighboring wells during handling of BAC plates for MTP rearraying or sequencing. So if both BACs were fully assembled, one could use their sequences to link BAC clusters under the condition that further evidence corroborates the connection. We identified inconsistently anchored BACs (termed ‘link BACs’) that showed stringent sequence overlaps (≥ 10% of the assembled length of either BAC) to two BACs B1 and B2 at the ends of different clusters. We required BACs B1 and B2 to originate from the same FP contig and to be anchored within 1 cM of each other in the POPSEQ genetic map. If these criteria were met, we added an edge between B1 and B2 in the overlap graph. We did not add the link BAC itself to avoid introducing contaminant sequences from other parts of the genome. This step did not introduce branches or inconsistencies with genetic data. The number of clusters decreased to 4,486 (Table 3).

Step 7: Using singletons BACs to bridge gaps in FP contigs

In this step, we tried to find single BACs that can close gaps within FP contigs. We identified pairs BACs B1 and B2 that were located on the same FP contigs, but different clusters, and searched for a third B3 that had stringent sequence overlap (≥ 10% of the assembled length of either BAC) to both B1 and B2. We required that B3 was a singleton (i.e., a cluster of size 1) and was within 3 cM of both B1 and B2 and the POPSEQ genetic map. If these criteria, were fulfilled we added edges B3<->B1 and B3<->B2. No branches or inconsistencies with the POPSEQ map were introduced in this step. This step resulted in the merging of two adjacent clusters and the incorporation of one singleton (Table 3).

Step 8: Using FP information and BioNano data

We searched for links between two BAC clusters that were part of the same FP contig and that were supported by alignments to a single BioNano contig. We searched the BioNano map for links between clusters as described in the section ‘Adding links with permissive overlap criteria, but support by the BioNano map’. We required the alignments of connected clusters to be no farther apart than 300 kb and that the corresponding BACs came from the same FP contig and were located within 300 kb in the FP map. Moreover, the order and orientation in the FP contig and the BioNano map were required to be consistent with each other. If these criteria were fulfilled, we added an edge between the BACs at the abutting end of the two connected clusters. This step introduced inconsistencies to the POPSEQ map that were removed by deleting all newly inserted edges in the affected clusters. This step reduced the number of clusters from 4,485 to 4,390 (Table 3).

Step 9: Adding BACs previously considered as inconsistently anchored

We searched for BACs who (i) were flagged as inconsistently anchored because of the standard deviation of the genetic coordinates of the Morex WGS aligned to them was larger than 3 cM, (ii) had stringent overlaps to non-singleton BACs. We required that all Morex WGS contigs aligning to these BACs originated from the same chromosome. We added these BACs and edges leading to them to the overlap graph. This step introduced branches to the overlap graph, which were removed by deleting the newly added BACs in branched clusters. This step resulted in the incorporation of 75 additional BACs into the overlap graph (Table 3).

Step 10: Using BAC overlap information and BioNano data

In this step, we used BAC sequence overlap information and BioNano map data to add edges to the overlap graph. We found potential connections between clusters as detailed in the section ‘Adding links with permissive overlap criteria, but support by the BioNano map’. If the two BACs B1 and B2 at the adjoining ends of the two linked clusters were within 3 cM of each other and the overlapping regions was (≥ 10% of the assembled length of either BAC), we added an edge between B1 and B2. This step did not introduce branches or inconsistencies with the genetic map. The updated graph consisted of 4,323 clusters (Table 3).

Step 11: Using FP information to bridge gaps

In this step, we aimed to use the BioNano map to close gaps between two BACs B1 and B2 that are near to each other in the physical map and were expected to overlap with a common BAC B3 between them (layout: B1 ->B3 ->B2) based on fingerprinting results, but their sequence assemblies failed to do so, resulting in a short gap between B1 and B2. Towards this purpose, we identified pairs of BACs B1 and B2 that (i) were on the same chromosome less than 3 cM part and (ii) located at the ends of two different overlap clusters and (iii) came from the same FP contigs, (iv) were separated by less than 300 kb in the FPC map with a single BAC B3 between them in the FPC map. Such cases may occur if both B1 and B2 were expected to overlap with B3 according to FPC information, but either the overlapping regions could not be detected in the alignment of the sequence assemblies because of low assembly quality or because of BAC mix-ups during fingerprinting, re-arraying of MTP clones or sequencing library preparation, so that B1 and B2 were separated by a gap in the overlap graph. We added an edge between B1 and B2 if the following conditions were fulfilled: (i) the two clusters of B1 and B2 could be aligned to the same contig of the BioNano map, (ii) the aligned regions were less than 300 kb apart in the BioNano map and (iii) the orientation of the BioNano contigs and the overlap clusters were consistent. This step did not introduce branches or inconsistencies with genetic data. This step decreased the number of clusters from 4,323 to 4,259 (Table 3).

Step 12: Adding links supported by BAC end sequences and the BioNano map

We identified BACs link supported by BAC end sequences and the BioNano map as described in Step 4, but did not require the connected BACs to come from the same FP contig. Added links meeting the criteria to the overlap graph did not create branches or inconsistencies. The final graph consisted of 80,010 BACs in 4,251 clusters and 3,938 singleton BACs (Table 3).

### Construction of non-redundant sequences of BAC overlap clusters

A non-redundant sequence was constructed for each BAC cluster by detecting and removing sequence overlaps between neighboring BACs using an iterative procedure. In the initial step, the complete sequence of the largest sequence scaffold among the assemblies of all BACs in a cluster was added to the set of visited BAC sequence scaffolds, all other sequence scaffolds were part of the set of unvisited BAC sequence scaffolds. The set of unvisited sequence scaffolds was then aligned to the visited sequence scaffolds with megablast^27^ with a word size of 44, accepting only high-scoring pairs with an alignment length≥500 bp and an alignment identity≥99.5 bp. Alignments between two sequence scaffolds from BACS B1 and B2 were only allowed if B1 and B2 were separated in the minimum spanning tree of the cluster by no more than 10 BACs. Regions contained in alignments to visited scaffolds satisfying these criteria were subtracted from the unvisited sequence scaffolds using BEDTools^40^. Sequence scaffolds that were composed of less than 500 proper nucleotides (ACGT characters) after subtraction were discarded. The largest sequence scaffold among the unvisited scaffolds was moved from the set of unvisited to the set of visited scaffolds. These steps of alignment, redundancy removal and selection of the largest unvisited scaffold were repeated until no unvisited scaffolds remained. Finally, stretches of N characters at the ends of non-redundant fragments of sequence scaffolds were trimmed with an AWK script. After these procedures had been carried out for all BAC clusters, the resultant non-redundant sequences were written into a single FASTA file (Data Citation 37).

### Construction of a high-resolution GBS map of the Morex x Barke population

At this stage, we constructed a high-resolution linkage map from GBS data using the non-redundant sequence as a reference for read alignment. This map was used to derive orientations of BAC overlap clusters in the Hi-C map (see ‘Orienting clusters by Hi-C and GBS’) and to validate the order of clusters in the Hi-C map (see ‘Technical Validation’). GBS libraries of 2,398 recombinant inbred lines of the Morex x Barke lines were constructed using published protocols^46,50^ and subjected to Illumina or IonTorrent sequencing (Data Citation 38). Adapters were trimmed from GBS reads with cutadapt^51^ version 1.8.1. Reads shorter than 30 bp after trimming were discarded. Trimmed reads were mapped to the non-redundant sequence of BAC clusters with BWA^45^ mem version 0.7.12. The resultant alignment files were converted to BAM format with SAMtools^52^ (version 0.1.19), sorted with Novosort (Novocraft Technologies Sdn Bhd, Malaysia, http://www.novocraft.com/) and merged into a single BAM files with Picard (version 1.128, http://broadinstitute.github.io/picard/). Multi-sample SNP calling was performed with FreeBayes^53^ using the parameters ‘-i -X -u -n 2 -$ 5 -e 2 -m 20 -q 20 --min-coverage 500 -G 200 -F 1 -w --genotype-qualities --report-genotype-likelihood-max’. The resulting VCF file was filtered with an AWK scripts (Text S3 of Mascher *et al.*^46^). Only bi-allelic SNP with a quality score≥40 were considered. Homozygous genotype calls were set to missing if their read depth was below 2 or their quality score below 20. Heterozygous genotype calls were ignored. Variants with more than 50% missing data or a minor allele frequency below 30% were discarded. The filtered SNP-by-individual matrix was imported into the R statistical environment^41^ for further processing. After removing samples with less than 6,000 successful genotype calls, the final marker-by-individual matrix was constructed by discarding SNPs with more than 10% missing data. Genetic map construction was done with MSTMap^47^ with a *P*-value cut off of 1×10^−60^ using the population type ‘RIL8’. The final map included genotypic data from 1,613 individuals at 2,637 variant positions (Table 5, Data Citation 39).

### Hi-C map construction

Hi-C map construction comprised the steps (i) data alignment to the non-redundant sequence, (ii) ordering and (iii) orienting BAC clusters using Hi-C link information.

#### Alignment of Hi-C data to restriction fragments

A BED file representing all intact HindIII restriction fragments≥100 bp within in the non-redundant sequence was constructed using a custom AWK script. Whole genome shotgun reads^4^ of barley cv. Morex corresponding to ~14x whole genome coverage were aligned to non-redundant sequence with BWA mem 0.7.12 (ref. 45), converted to BAM format with SAMtools^52^. Duplicate removal and sorting were done with Novosort. The coverage of the non-redundant sequence with WGS reads was calculated with SAMtools^52^ using the command ‘depth –Q 20 –q 10’ and written into a BED file. This file was used to calculate the average coverage of each HindIII fragment using the BEDTools^40^ command ‘map’. Fragments with an average coverage below 7 or above 21 were discarded.

Paired-end reads^9^ (Data Citation 40) obtained using the Hi-C and TCC protocols^9,54^ as described in ref. 42 were trimmed using cutadapt^51^ version 1.8.1 using as the adapter sequence the ‘extended’ NheI restriction site (AAGCTAGCTT) created by ligating two blunted HindIII fragments^9^. Trimmed read pairs were mapped as single ends to the non-redundant sequence using BWA mem version 0.7.12 (ref. 45) with parameters ‘-M –P –S’ and then converted to BAM format with SAMtools^52^. After duplicate removal with Novosort (Novocraft Technologies Sdn Bhd, Malaysia, http://www.novocraft.com/), BAM files were sorted by read name to group the two mates of a pair together. Hi-C mapping information was then converted from BAM to BED format and assigned to HindIII restriction fragments with BEDTools^40^ using the command ‘pairtobed -bedpe –type both ‘ requiring both mates of a pair to have mapping quality≥10. A custom AWK script was used to calculate the size of sequence fragments that read pairs originated from based on the distance of mapped ends to the next HindIII restriction site. After discarding fragments with size≥500 bp, read pairs linking two different clusters (Hi-C links) were tabulated using standard UNIX tools (AWK, sort, uniq) and the link counts for each cluster pair were imported into R^41^.

#### Ordering scaffolds by Hi-C

Clusters whose non-redundant sequence was less than 30 kb or which had less than 20 restriction fragments were not used for making the Hi-C map. Scaffold ordering with Hi-C data was done using a custom R implementation of the algorithm outlined in Burton *et al.*^10^. First, the Hi-C link information was entered into graph structure using the R package ‘igraph’ (http://igraph.org/r/). The graph was composed of nodes representing the clusters and of edges representing Hi-C links between them. The edge weights were set to –log_10_(number of Hi-C links). Only links between clusters anchored genetically to the same chromosome within 15 cM of each other were considered. For each of the seven largest connected components (corresponding to the seven chromosomes of barley), a minimum spanning tree was calculated with Prim’s algorithm^49^ as implemented in igraph. This resulted in a backbone map into which further nodes (clusters) were inserted so as to minimize the additional weight incurred by each node insertion. Subsequently, the 2-opt heuristics and single node relocation as used in the MSTMap algorithm for genetic mapping^47^ were applied to incorporate local perturbations that reduce the weight sum of the initial solution. The resultant paths of each connected component (chromosome) were oriented from short to long arm by comparison to the POPSEQ genetic map.

#### Orienting clusters by Hi-C and GBS

To orient clusters relative to the telomeres of the long and short chromosome arm, clusters were divided into bins of 300 kb size that were ordered by Hi-C as described above. If a cluster comprises several bins, the scaffold orientation can be inferred from the order of its constituent bins in the global Hi-C map of all 300 kb bins, which is oriented on a chromosome scale (from short to long arm) by comparison to the genetic map as described above. Local inversions may arise in the Hi-C map of the bins because of the reduced accuracy of Hi-C mapping when smaller intervals are used to aggregate Hi-C link information. To correct inverted orientations in the bin map, we checked how the relative order of a cluster C and its two adjacent clusters was correlated with that of their constituent bins. If the correlation coefficient was negative, the orientation of cluster C was reversed. If no HIC orientation could be determined, but orienting clusters was possible using GBS marker information, this information was used instead. The orders and orientation of sequence clusters are given in Data Citation 41.

### Construction of pseudomolecule sequences

We constructed a FASTA file containing a single entry for each barley chromosome (a ‘pseudomolecule’) and an additional entry combining all sequence not anchored to chromosomes. Prior to the construction of pseudomolecules, we (i) identified genes incomplete or missing in the non-redundant sequence, but represented by (a) BAC sequence that had been excluded from the construction of the non-redundant sequence, or by (b) Morex WGS contigs^4^; and (ii) performed a final scan for contaminant sequences.

#### Identification of additional gene-bearing sequences

The sets of (i) barley high-confidence (HC) genes annotated on the WGS assembly of cv. Morex^4^ and (ii) barley full-length cDNA (fl-cDNA) sequences^55^ were aligned with GMAP^56^ version 2014-12-21 to (a) the set of all BAC assemblies, (b) Morex WGS contigs^4^ and (c) the non-redundant sequence.

First, we identified genes (as represented by the HC genes or fl-cDNAs) whose best alignment to the set of assembled sequences of all BACs in clusters (as opposed to BACs excluded from the overlap analysis) represented at least 5% more of their coding sequence than their best alignment to the non-redundant sequence. Such cases arise if during the iterative construction of the non-redundant sequence, a sequence contig (or scaffold) C1 that breaks within a gene G is chosen before a contig C2 that contains a larger part of G than C1, but the total length of C1 is larger than that of C2. To amend such situations, we added contigs of type C2 to the non-redundant sequence and removed contigs of the non-redundant sequence that had previously represented the sequence now covered by C2. Towards this purpose, we aligned the sequence of each C2-type contig C to the non-redundant sequence of its BAC cluster of origin with megablast^27^ using a word size of 44 and considering only high-scoring pairs with an alignment length≥500 bp and an alignment identity≥99.5%. Regions of the old non-redundant sequence covered by C (as determined by commands of BEDTools^40^ suite) were removed and contig C was added instead. This procedure was performed for each C2-type contig.

Next, we queried the GMAP alignments for genes that had no alignments to the non-redundant sequence, but were represented either in (a) the Morex WGS contigs or in (b) sequences of BACs excluded from the overlap analysis. We considered sequence of type (a) and (b) as ‘additional gene-bearing sequences’. We aligned these additional gene-bearing sequences to the non-redundant sequence with megablast^27^ using a word size of 44 and considering only high-scoring pairs with an alignment length≥500 bp and an alignment identity≥99.5%. Regions covered by the non-redundant sequence under these alignment criteria were subtracted from the additional gene-bearing sequences and sequence fragments with a length≥500 bp were added to the non-redundant sequence.

#### Final contamination removal

We identified regions in the non-redundant sequence that were not covered by whole-genome shotgun reads of cv. Morex. Alignment of WGS reads and read depth calculation were done as described in the section ‘Alignment of Hi-C data to restriction fragments’. Regions of the non-redundant sequence not covered by Morex WGS reads and with a length≥500 bp were extracted using UNIX command line tools and BEDTools^40^ (command ‘getfasta’). The extracted sequences were aligned to the NCBI NT database with megablast^27^ using a word size of 44 and requiring the high-scoring pairs to have a length of at least 100 bp and an alignment identity≥80%. We retained only hits whose description in the NCBI NT database did not match the following regular expression (R syntax) representing a list of common and taxonomic names of plant species:

‘Hordeum|Triti|Populus|Aegilops|Avena|Alnus|A\\.squarrosa|Morus|Nelumbo|Brassica|Cucumis|Citrus|Camelina|Fragaria|Lotus|Tarenaya|Spartina|Eucommia|Sorghum|Corylus|Theobroma|Phaseolus|Barley|Trifolium|Elymus|Brachypodium|Beta vulgaris|Ricinus|Licania|Phoenix|H\\.vulgare|Pyrus|Malus|Prunus|Saccharum|Hypericum|Wheat|Oryza|hloroplast|Secale|Vitis|Quercus’

Regions overlapping the BLAST hits passing these filters were cut from the non-redundant sequence with BEDTools^40^ (command ‘subtract’). Sequences shorter than 500 bp after the removal of contaminant sequences were discarded. This step removed 5 Mb (0.1%) of the assembled sequence.

#### Construction of pseudomolecule sequences for chromosome 1H—7H and chrUn

We constructed pseudomolecules of the seven barley chromosomes by placing the sequence fragments of single BAC assemblies that constitute the non-redundant sequence according to the Hi-C map positions of the BAC overlap clusters these fragments belong to. Sequences not anchored by Hi-C were placed on chrUn (‘chromosome unassigned’). The order of clusters was taken from the Hi-C map. BACs within the same cluster were ordered according to the minimum spanning tree of the BAC overlap graph of the cluster and oriented relative to the telomeres using the Hi-C orientation of the cluster if available. The relative order of sequence fragments originating from the same BAC bin (see section ‘Construction of the BAC overlap graph’) could not be determined so that the placement of sequences within a BAC bin (average size: 70 kb) is arbitrary. ChrUn is composed of (i) sequence fragments originating from BAC overlap clusters not placed in the Hi-C map, or (ii) gene-bearing fragments of BAC sequences and Morex WGS contigs selected in addition to the non-redundant sequence (see section Identification of additional gene-bearing sequences). A gap of 100 N characters was inserted between adjacent sequence fragments. Pseudomolecules of all chromosomes and chrUn were combined into a single FASTA file (Data Citation 42). To accommodate limitations of the Sequence/Alignment Map format (see Usage Notes) split pseudomolecules with a size below 512 Mb were constructed by breaking pseudomolecules arbitrarily at breaks between sequence contigs (Data Citation 43, Data Citation 44). A BED file indicating the placement of BAC sequence fragments, Morex WGS contigs and intercalating gaps in the (split) pseudomolecules is available for download (Data Citation 45, Data Citation 46).

A tabular summary of the positional information incorporated into pseudomolecules is given in Data Citation 41.

### Masking of residual redundancy

Residual redundancy arising from undetected overlaps between adjacent BACs was detected and masked by aligning the pseudomolecules sequence to itself with megablast^27^. Genomic intervals contained in BLAST hits with a length≥5 kb and an identity≥99.8% were considered as potentially redundant (PR) regions. PR regions were classified to decide which sequence of a redundant pair to mask: (i) PR regions assigned to chromosomal pseudomolecules (as opposed to chrUn), but having BLAST hits only to other chromosomes were considered as originating from chimeric BAC assemblies incorporating unrelated sequences from different chromosomes and masked with Ns; (ii) an analogous procedures was used to find intrachromosomal chimeras based on Hi-C map information; (iii) PR regions on chrUn that had alignments to regions on chromosomal pseudomolecules were masked, (iv) for other PR regions one sequence of a redundant pair was chosen arbitrarily. Positions of masked regions on the (split) pseudomolecules were written into a BED file (Data Citation 47, Data Citation 48). Masking was done with BEDTools^40^ (command ‘mask’) overwriting nucleotides in redundant intervals with N characters. Masked versions of the (split) pseudomolecules are provided as Data Citation 49, Data Citation 50).

### POPSEQ genetic map based on pseudomolecule sequence

After the construction of the map-based reference sequence, we constructed an updated high-resolution genetic map of the Morex x Barke population to validate the order of genetic map in the reference sequence. Raw reads (see section ‘Construction of the updated POPSEQ map of the Morex x Barke mapping population’) were aligned to the barley pseudomolecules with BWA mem (version 0.7.12)^45^. Checking mated mapped paired reads, sorting, conversion to BAM format and marking of duplicate read pairs were done with PicardTools version 2.300 (http://broadinstitute.github.io/picard/). Variant detection and genotype calling were performed using GATK Toolkit version 3.3.0 (command ‘HaplotypeCaller’)^57^. A total of five RILs with >3% heterozygous variants were removed. A variant position was removed if more than 10% of all samples were called heterozygous, there were more than 80% missing data, or the minor allele frequency (in the non-missing data) was smaller than 5%. SNP information was aggregated at the contig level to derive consensus genotype blocks with false discovery rate calculated based on the quality of each variant call in the block. High-confidence genotype blocks were obtained based on a Bonferroni correction threshold. Given the fact that the length of crossover tracts is significantly larger than that of non-crossover tracts and non-crossover tracts would enlarge the genetic distance artificially, we only retained high-confidence genotype blocks with more than 1 Mb tract length, which are likely to be derived from crossovers. Representative non-redundant genomic variants of high-confidence genotype blocks were extracted and used for the construction of a high-resolution map through MSTMap^47^. We further anchored all remaining markers to the genetic map by the C program ‘canchor’^5^. The final POPSEQ map consisted of 9,012,742 SNP variants defined on the pseudomolecule sequence Data citation 51).

### Representation of full-length cDNAs

The representation of gene models in the whole-genome genome assembly of barley cv. Morex^4^ and in the pseudomolecules was compared by aligning a set of 22,651 publicly available full-length cDNAs^55^ to the assemblies using the GMAP splice aligner software^56^. The GMAP alignment output was then filtered. If a full-length cDNA had multiple hits, only the hit with the highest % identity was considered. Hits were further filtered by identity (≥ 98%) and coverage (≥ 95%). This resulted in a set of hits representing genes recovered intact on a single genomic contig/chromosome.

### Code availability

R and shell source code for the construction of the BAC overlap graph and the Hi-C map is provided as Data Citation 52. Code can be re-used under the terms of the MIT license.

## Data Records

BAC sequence raw data was submitted to the European Nucleotide Archive (ENA) (Data Citation 1, Data Citation 2, Data Citation 3, Data Citation 4, Data Citation 5, Data Citation 6, Data Citation 7, Data Citation 8, Data Citation 9, Data Citation 10, Data Citation 11, Data Citation 12, Data Citation 13, Data Citation 14, Data Citation 15, Data Citation 16, Data Citation 17, Data Citation 18, Data Citation 19, Data Citation 20, Data Citation 21, Data Citation 22, Data Citation 23, Data Citation 24, Data Citation 25, Data Citation 26, Data Citation 27). BAC assemblies were submitted to ENA or NCBI (Data Citation 28, Data Citation 29). Raw data for POPSEQ (Data Citation 35), GBS (Data Citation 38) and Hi-C mapping (Data Citation 40) were submitted to ENA. Processed datasets are accessible as Digital Object Identifiers (DOIs) in the Plant Genomics and Phenomics Research Data Repository^58^ (Data Citation 30, Data Citation 31, Data Citation 32, Data Citation 33, Data Citation 34, Data Citation 36, Data Citation 37, Data Citation 39, Data Citation 41, Data Citation 42, Data Citation 43, Data Citation 44, Data Citation 45, Data Citation 46, Data Citation 47, Data Citation 48, Data Citation 49, Data Citation 50, Data citation 51, Data Citation 52). DOIs were registered with e!DAL^59^.

## Technical Validation

### Collinearity between genetic maps and pseudomolecules

**Figure 2:**
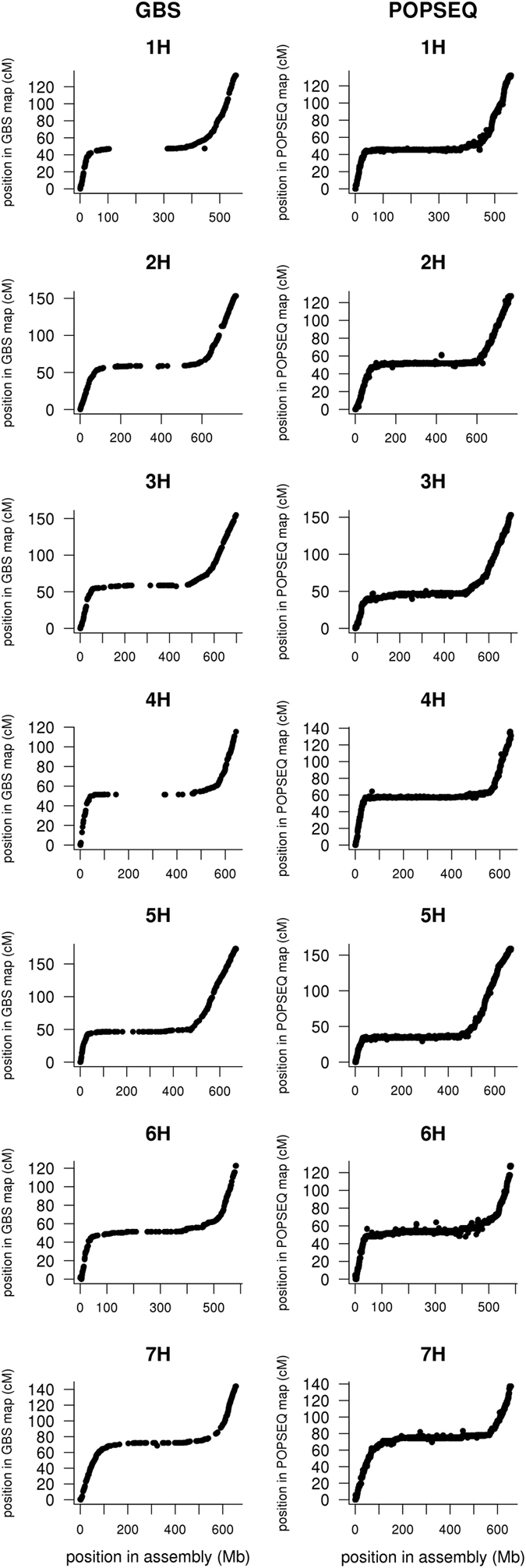
Collinearity between the Hi-C map and two genetic maps. The positions of genetic markers (x-axis) are plotted against their genetic positions (y-axis) in a GBS map (top row) and a POPSEQ map (bottom row) of the Morex x Barke recombinant inbred lines.

To validate the order of scaffolds in the Hi-C map, we compared the order of genetic marker loci in the Hi-C-derived pseudomolecules to their positions in linkage maps. First, we used genotyping-by-sequencing (GBS)^11,50^ to type single-nucleotide polymorphisms (SNPs) segregating in a bi-parental population comprising 2,398 recombinant inbred lines (RILs). A total of 2,637 SNPs were detected by aligning GBS reads and calling variants and genotypes using a previously published pipeline^46^. Second, we reanalysed WGS re-sequencing data of a subset of the same population (POPSEQ data) comprising 90 RILs. Construction of a framework linkage map and insertion of additional markers were performed essentially as described by Chapman *et al.*^44^ A dot plot comparison of physical and genetic SNP positions revealed that marker orders were highly collinear between the pseudomolecules and both the GBS and POPSEQ map of the Morex x Barke population (Fig. 2).

### Collinearity between a cytogenetic map and the pseudomolecule of chromosome 3H

**Figure 3:**
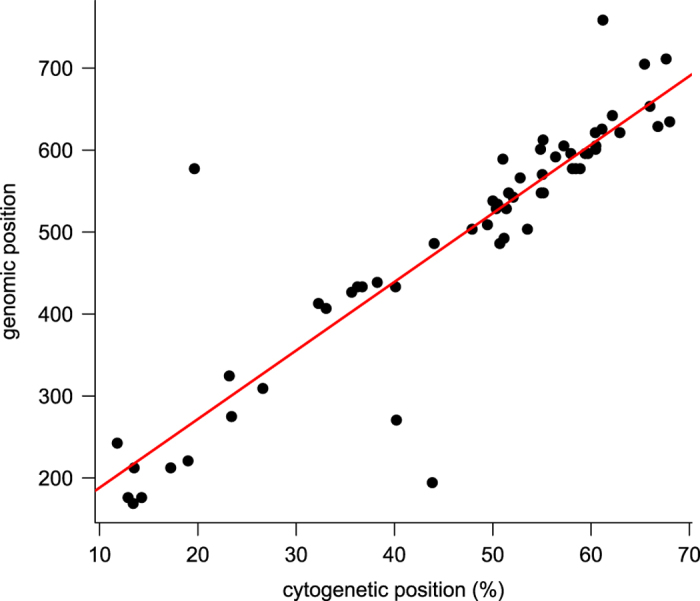
Collinearity between the Hi-C map and a cytogenetic map of chromosome 3H. Dots mark the positions of probes in the cytogenetic map (x-axis) and the Hi-C-derived pseudomolecule (y-axis). A linear regression line (red) was fitted with the R function lm(). Note that cytogenetic data is not available for distal regions because probes were designed only for non-recombining peri-centromeric regions^61^.

We could not validate the order of BAC overlap clusters in the large peri-centromeric regions because of severely repressed recombination^3,60^. Therefore, we compared the order of probes mapped by fluorescence *in-situ* hybridization to chromosomal locations on chromosome 3H and their corresponding sequences in the pseudomolecule of 3H. Since probes were derived from BAC sequences associated with physical contigs, their position from the reference sequence could be determined from the BAC overlap graph. The comparison showed that the cytogenetic and Hi-C maps were highly collinear in peri-centromeric regions of chromosome 3H (Fig. 3).

### Representation of full-length cDNAs

To assess the completeness of our assembly, we checked for the presence of high-confidence transcript sequences. The representation of gene models in the whole-genome shotgun assembly of barley cv. Morex^4^ and in the map-based reference assembly was compared by aligning a set of 22,651 publicly available full-length cDNAs^55^ of barley cv. ‘Haruna Nijo’. After aligning and filtering, 18,062 (79.74%) intact full-length cDNAs were found in the pseudomolecules, whereas only 10,496 (46.33%) were recovered in the whole-genome assembly. This increase in the number of correctly represented full-length cDNAs vindicates the effort invested in the map-based assembly. Nevertheless, a significant proportion of genes remain fragmented even in the pseudomolecule assembly (20.26%), and presumably these largely represent difficult to assemble genes that contain e.g., microsatellites, long homopolymer stretches and other difficult features, and/or form part of complex gene families that are difficult to resolve. It is likely that only longer read technologies such as Pacific Biosciences (http://www.pacb.com) or Oxford Nanopore (https://www.nanoporetech.com) will be able to resolve these more difficult cases. Further results on gene space completeness based on an automated gene annotation of the pseudomolecules, and on the representation of repetitive elements are described elsewhere^42^.

## Usage Notes

**Figure 4:**
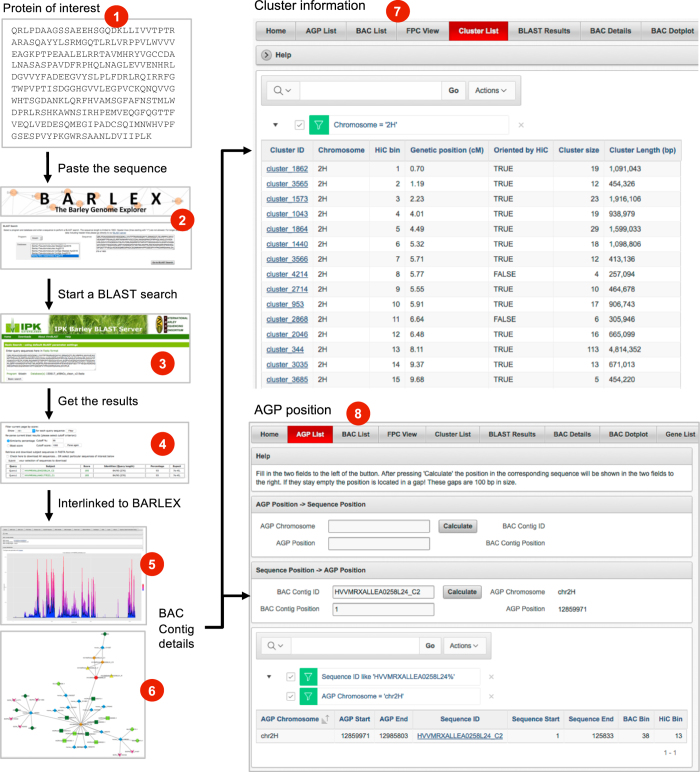
Accessing sequence and positional information with the barley genome explorer (BARLEX). The barley pseudomolecule data was imported into BARLEX, where it is directly linked to the IPK Barley BLAST server. Users can paste a nucleotide or amino acid sequence (1) into the BARLEX input query form and select reference database such as pseudomolecules sequence, the set of all BAC assemblies or annotated genes (2). The sequence is then transferred to the IPK barley BLAST Server (3). The web page with the BLAST results (4) contains references to BARLEX information pages for different structural units (BAC sequence contigs, BAC, BAC cluster, chromosomal Hi-C map). For example, the pages of BAC sequence contigs visualize the repeat content based on genome-wide *k*-mer histograms (5) and are linked to a graph-based visualization (6) of the entire BAC assembly. Summary statistics and positional information of BAC clusters are presented in tables that can be searched, sorted and subsetted using user-defined criteria (7). Users can convert pseudomolecule coordinates (AGP positions) to intervals in the underlying BAC sequence assemblies (8).

Positional information for BAC sequences, physical contigs and WGS contigs can be accessed via the barley genome explorer BARLEX (Fig. 4). BLAST searches against the barley pseudomolecules can also be carried out in BARLEX. We note that processing BAM files with short read alignments to the full pseudomolecules with commonly used tools such as SAMtools^52^ or BEDTools^40^ may not work as expected because of restrictions on the chromosome size (512 Mb) for indexing file in Sequence Alignment/Map (SAM) format^52^. To circumvent this issue, we have split the pseudomolecules into two part and provide (i) a FASTA file with split pseudomolecules (Data Citation 44) along the with the intact sequences and (ii) a BEDfile to convert between full and split pseudomolecule coordinate (Data Citation 43) Alternatively, the CRAM format (https://samtools.github.io/hts-specs/CRAMv3.pdf) may be used instead of the BAM format. We note that the orientation of sequence contigs within individual BACs in the pseudomolecules is arbitrary, thus the order and orientation of sequences in the pseudomolecules is accurate only up to resolution of ~100 kb.

## Additional Information

**How to cite this article**: Beier, S. *et al.* Construction of a map-based reference genome sequence for barley, *Hordeum vulgare* L. *Sci. Data* 4:170044 doi: 10.1038/sdata.2017.44 (2017).

**Publisher**’**s note**: Springer Nature remains neutral with regard to jurisdictional claims in published maps and institutional affiliations.

## Supplementary Material



## Figures and Tables

**Table 1:** BAC assembly and anchoring statistics.

**MTP chromosome**	**no of. BACs in MTP**	**no. of sequenced BACs**	no. of anchored BACs*	**average no. of sequences**	**average N50 (kb)**
**1H**	6,993	6,983 (99.9%)	6,410 (91.8%)	7.6	81.2
**2H**	9,061	8,969 (99.0%)	8,195 (91.4%)	9.9	104.5
**3H**	8,841	8,807 (99.6%)	8,303 (94.3%)	7.7	87.5
**4H**	8,314	8,306 (99.9%)	7,783 (93.7%)	6.7	91.2
**5H**	8,426	8,358 (99.2%)	7,573 (90.6%)	9.7	72.2
**6H**	8,305	7,886 (95.0%)	6,476 (82.1%)	7.4	70.7
**7H**	8,576	7,970 (92.9%)	6,842 (85.8%)	8.5	65.5
**‘0H’**†	8,256	8,031 (97.3%)	6,714 (83.6%)	7.6	83.6
**Non-MTP**	—	21,765	20,397 (93.7%)	14.5	33.7
**Total**	66,772	87,075	78,693 (90.4%)	9.8	70.3

^*^Number and percentage of BAC clones that have been assigned genetic positions in the POPSEQ map.

^†^BAC clones in physical contigs that had not been assigned to chromosomes.

**Table 2:** Summary statistics of the updated POPSEQ map of the Morex WGS assembly.

**Chromosome**	**No. of anchored WGS contigs**	**Length of anchored WGS contigs (Mb)**
**1H**	74,184	123.7
**2H**	130,436	202.6
**3H**	119,131	187.6
**4H**	96,642	170.6
**5H**	117,314	177.8
**6H**	121,384	168.4
**7H**	132,085	190.2
**Total**	791,176	1220.9

**Table 3:** Cluster summary statistics after each step of the BAC overlap graph construction.

**Step**	Datasets*	**Clusters**	**BACs in clusters**	**Singleton BACs**	**Excluded BACs**	**Cluster N50**†	**Average cluster size**‡
1	BAC, FPC	9,637	71,828	13,211	2,036	21	12.9
2	BAC	4,890	79,871	4,002	3,202	60	38.3
3	BAC, OM	4,843	79,884	3,989	3,202	61	38.8
4	FPC, BES, OM	4,653	79,884	3,989	3,202	65	41.2
5	FPC, BES	4,562	79,908	3,965	3,202	66	41.7
6	BAC, OM	4,486	79,918	3,955	3,202	66	42.4
7	FPC, BAC	4,485	79,919	3,954	3,202	66	42.4
8	FPC, OM	4,390	79,919	3,954	3,202	66	43.0
9	exBAC	4,382	80,010	3,938	3,127	66	43.1
10	BAC, OM	4,323	80,010	3,938	3,127	67	43.8
11	FPC, OM	4,259	80,010	3,938	3,127	69	45.2
12	BES, FPC	4,251	80,010	3,938	3,127	69	45.2

^*^Datasets used in each step (BAC, BAC sequence overlap; FPC, physical map; OM, optical map; BES, BAC end sequences; exBAC, previously excluded BAC assemblis. Consistency with the POPSEQ genetic map was checked in each step.

^†^An N50 value *N* indicates that half of all clusters contain at least *N* BACs.

^‡^Arithmetic mean of the number of BACs per cluster.

**Table 4:** Final cluster statistics.

	**1H**	**2H**	**3H**	**4H**	**5H**	**6H**	**7H**	**Un**
**Number of clusters**	389	605	324	415	549	768	943	242
**Number of singletons**	65	214	74	78	173	167	162	1190
**Assembly length (Mb)**	562.8	785.5	704	655.5	687.8	600.2	663.8	130.6
**Length in clusters (Mb)**	555.9	760.3	695.8	648.4	668.2	581.1	646	28.9
**Length in singletons (Mb)**	6.9	25.1	8.3	7.1	19.5	19.1	17.7	101.7
**N50 (Mb)**	2.5	2.1	3.6	2.5	2.0	1.1	1	0.1

**Table 5:** Summary statistics of the GBS map.

**Chromosome**	**No. of SNPs**	**No. of bins**	**Map length (cM)**
**1H**	346	195	133.3
**2H**	383	231	153.2
**3H**	385	231	154.9
**4H**	237	135	115.5
**5H**	474	265	173.3
**6H**	362	188	122.7
**7H**	450	253	143.9
**total**	2,637	1,498	996.8
